# SARS‐CoV‐2 infections in patients enrolled on the Children's Oncology Group standard‐risk B‐cell acute lymphoblastic leukemia trial, AALL1731

**DOI:** 10.1002/jha2.697

**Published:** 2023-05-10

**Authors:** Caitlin W. Elgarten, John A. Kairalla, Joel C. Thompson, Tamara P. Miller, Cindy Wang, Susan Conway, Mignon L. Loh, Elizabeth A. Raetz, Sumit Gupta, Rachel E. Rau, Anne Angiolillo, Karen R. Rabin, Sarah Alexander

**Affiliations:** ^1^ Department of Pediatrics Division of Oncology Children's Hospital of Philadelphia Philadelphia Pennsylvania USA; ^2^ Department of Biostatistics University of Florida Gainesville Florida USA; ^3^ Department of Pediatrics Division of Hematology/Oncology/Bone Marrow Transplant Children's Mercy Hospital University of Missouri ‐ Kansas City Kansas City Missouri USA; ^4^ Pediatric Hematology/Oncology Children's Healthcare of Atlanta ‐ Egleston Atlanta Georgia USA; ^5^ Ben Towne Center for Childhood Cancer Research Seattle Children's Research Institute and Department of Pediatrics Seattle Children's Hospital University of Washington Seattle Washington USA; ^6^ Laura and Isaac Perlmutter Cancer Center at NYU Langone New York New York USA; ^7^ Division of Hematology/Oncology Hospital for Sick Children Toronto Ontario Canada; ^8^ Pediatric Hematology/Oncology Baylor College of Medicine Houston Texas USA; ^9^ Center for Cancer and Blood Disorders Children's National Medical Center Washington District of Columbia USA

**Keywords:** B‐ALL, pediatrics, SARS‐CoV‐2

## Abstract

Hematologic malignancy is a risk factor for severe coronavirus disease 2019 (COVID‐19) in adults; however, data specific to children with leukemia are limited. High‐quality infectious adverse event data from the ongoing Children's Oncology Group (COG) standard‐risk B acute lymphoblastic leukemia/lymphoma (ALL/LLy) trial, AALL1731, were analyzed to provide a disease‐specific estimate of SARS‐CoV‐2 infection outcomes in pediatric ALL. Of 253 patients with reported infections, the majority (77.1%) were asymptomatic or mildly symptomatic (CTCAE grade 1/2) and there was a single COVID‐19‐related death. These data suggest SARS‐CoV‐2 infection does not confer substantial morbidity among young patients with B‐lymphoblastic leukemia/lymphoma (B‐ALL/LLy).

**Abbreviations**
B‐ALL/LLy: B‐lymphoblastic leukemia/lymphoma
COG: Children's Oncology Group
COVID‐19: coronavirus disease 2019
CRF: case report forms
DS: Down syndrome
NCI: National Cancer Institute

## 1 INTRODUCTION

Coronavirus disease 2019 (COVID‐19) causes substantial morbidity, particularly in high‐risk populations [1]. Early in the pandemic, cancer, and especially hematologic malignancy, was identified as a risk factor for hospitalization and death due to COVID‐19 in adults [2, 3]. Data on SARS‐CoV‐2 risk in younger patients with cancer are more limited: some studies describe a mild clinical course similar to children without cancer [4], whereas others report high rates of severe infection, hospitalization, and death [5, 6, 7]. Conflicting findings, as well as heterogeneity in cancer types included in these studies, limit the clinical utility of these data. Disease‐specific estimates of SARS‐CoV‐2 burden can address this knowledge gap and inform decision‐making in pediatric patients with SARS‐CoV‐2 infection.

AALL1731 is a phase 3 randomized clinical trial (NCT03914625) for children with National Cancer Institute (NCI) standard‐risk (SR) B‐lymphoblastic leukemia (B‐ALL), and children and young adults with stage 1–2 B‐lymphoblastic lymphoma (B‐LLy) and Down syndrome (DS) B‐ALL that opened in June 2019, and has accrued subjects near the expected rate despite the COVID‐19 pandemic. Children's Oncology Group (COG) protocols account for a substantial and representative proportion of children in the United States with cancer; therefore, data from this trial provide a unique, population‐based assessment of the spectrum of SARS‐CoV‐2 infections in pediatric B‐lymphoblastic leukemia/lymphoma (B‐ALL/LLy) [8].

## 2 METHODS

AALL1731 includes children 1–10 years old with newly diagnosed NCI SR B‐ALL. Patients with DS younger than 31 years old are eligible for this trial regardless of presenting white blood cell count, as are patients with B‐LLy. In March 2020, the protocol was amended to mandate reporting of all grades of SARS‐CoV‐2 infection, graded according to the CTCAE v5 grading schema for “infections and infestations‐other.” For all infectious adverse events (AEs) on the trial, there is a case report form (CRF) for additional data on the infectious pathogen, and real‐time review of infectious AEs by pediatric oncologists to resolve incomplete or ambiguous submissions [9].

All patients enrolled on AALL1731 from June 28, 2019 to December 31, 2021, who had a reported SARS‐CoV‐2 infection, were included in these analyses. Demographic and treatment data were abstracted from CRFs. Body mass index (BMI) percentiles were based on anthropomorphic measurement at the start of the reporting period in which SARS‐CoV‐2 infection was documented. Statistical comparisons were performed using Wilcoxon rank‐sum and Fisher's exact tests for continuous and categorical variables, respectively.

## 3 RESULTS

**FIGURE 1:**
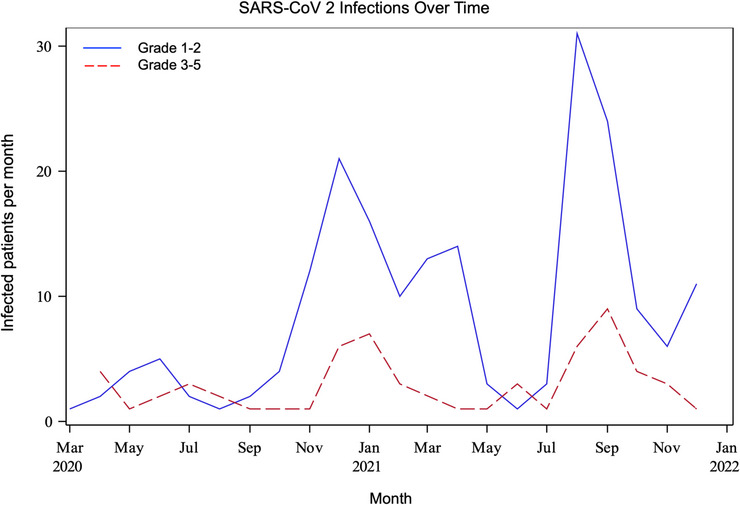
SARS‐CoV‐2 infections reported by month on AALL1731. Solid line represents infections with no or minimal symptoms (CTCAE grades 1 and 2), and dashed lines represent more severe infections requiring intervention or hospitalization (CTCAE grades 3–5).

As of December 2021, 253 patients at 113 centers experienced SARS‐CoV‐2 infections, accounting for 9.4% of all patients receiving protocol therapy during this time. Clinical characteristics are reported in Table 1. The substantial majority was less than 10 years old (96.4%), consistent with trial eligibility criteria, and the population was relatively enriched for patients treated in the United States (95.6% compared to 88% over overall trial participants treated in the United States). Case rates over time mimicked US trends, with relative peaks in December 2020 and September 2021 (Figure 1). Five patients had SARS‐CoV‐2 infection reported in non‐consecutive reporting periods suggestive of repeat infection or delayed clearance.

**TABLE 1:** Clinical characteristics of AALL1731 cohort with SARS‐CoV‐2 infection overall and according to severity of infection.

		Overall	Grades 1–2	Grades 3–5	*p*‐Value^a^
Total, *N*		253	195	58	
**Country of treating instution**					0.59
	United States	242 (95.7%)	185 (94.9%)	57 (98.3%)	
	Canada	10 (4.0%)	9 (4.6%)	1 (1.7%)	
	Australia	1 (0.3%)	1 (0.5%)	–	
**Female sex**		122 (48.2%)	94 (48.2%)	28 (48.2%)	1.00
**Age at diagnosis, years**					1.00
	Median (range)	4.6 (1.3–24.6)	4.6 (1.3–23.4)	4.4 (1.9–24.6)	
	Older than 10	9 (3.6%)	6 (3.1%)	3 (5.2%)	
**Race**					0.18
	Black or African American	20 (7.9%)	17 (8.7%)	3 (5.2%)	
	Other	13 (5.1%)	11 (5.6%)	2 (3.4%)	
	White	171 (67.6%)	135 (69.2%)	36 (62.1%)	
	Unknown	49 (19.4%)	32 (16.4%)	17 (29.3%)	
**Ethnicity**					0.18
	Hispanic or Latino	79 (31.2%)	55 (28.2%)	24 (41.4%)	
	Not Hispanic or Latino	144 (56.9%)	116 (59.5%)	28 (47.3%)	
	Unknown	30 (11.9%)	24 (12.3%)	6 (10.3%)	
**Insurance status**					0.69
	International	11 (4.3%)	10 (5.1%)	1 (1.7%)	
	US private/military	99 (39.1%)	78 (40.0%)	21 (36.2%)	
	US public	123 (48.6%)	92 (47.2%)	31 (53.4%)	
	Unknown (includes self‐pay)	20 (7.9%)	15 (7.7%)	5 (8.6%)	
**Trisomy 21**		19 (7.5%)	13 (6.7%)	6 (10.3%)	0.39
**B‐lymphoblastic lymphoma**		1 (0.4%)	1 (0.5%)	–	–
**Block of therapy**					0.29
	Pre‐maintenance	152 (60.0%)	119 (61.0%)	33 (56.9%)	
	Maintenance	89 (35.2%)	69 (35.4%)	20 (34.5%)	
	Follow‐up	12 (4.7%)	7 (3.6%)	5 (8.6%)	
**BMI (percentile for age) at start of block**					
	Median (range)	79.5 (0.2–100)	78.0 (0.2–100)	85.6 (3.0–99.7)	0.02
	<5th percentile	12 (4.7%)	11 (5.6%)	1 (1.7%)	0.3
	5th–85th percentile	126 (49.8%)	102 (52.3%)	24 (41.4%)	
	85th–95th	53 (20.9%)	40 (20.5%)	13 (22.4%)	
	>95th percentile	50 (19.8%)	35 (17.9%)	15 (25.9%)	
	Unknown	12 (4.7%)	7 (3.6%)	5 (8.6%)	

*Note*: Twelve cases were in follow‐up so no weight and height information reported.

^a^ Continuous tests are Wilcoxon rank‐sum, frequency tests are Fisher's exact.

The majority of patients (195, 77.1%) had CTCAE grade 1 or 2 infections, defined as either asymptomatic or mild infections requiring local intervention only. A minority, 58 (22.9%) patients, experienced more severe COVID‐19 (grades 3–5). One patient died with COVID‐19. This individual had multiple prior infectious complications of therapy including sepsis, meningitis, and acute hypoxic respiratory failure and had been removed from protocol therapy after induction. The child was diagnosed with SARS‐CoV‐2 infection via polymerase chain reaction (PCR) while receiving interim maintenance chemotherapy and died 3 weeks later with respiratory failure. Two patients experienced grade 4 infection, defined as “life threatening,” both while in maintenance. The remaining patients were grade 3, requiring or prolonging hospitalization but not immediately life‐threatening. Another grade 3 or higher infectious AE was reported within 7 days of grade 3–5 SARS‐CoV‐2 infection in 28 instances, including febrile neutropenia in 20, lung infection in two, enterocolitis in four, and sepsis in four.

Univariable associations with severe disease are shown in Table 1. Patients with severe infection were more likely to have a treatment delay of greater than 14 days in delivery of the next cycle of chemotherapy (43.4% vs. 18.1%, *p* ≤ 0.001). The majority (73.7%) of these delays were prior to the start of maintenance. The impact of this delay on outcomes is unknown as the trial is ongoing.

DS did not appear to be a risk factor for infection or for severe disease. Of patients with DS, a similar proportion (19/160, 11.9%) had a SARS‐CoV‐2 infection reported. Patients with DS accounted for 10.3% of those with severe infection compared to 6.7% without (*p* = 0.39).

## 4 DISCUSSION

The COVID‐19 pandemic has unique implications for children with ALL/LLy. While their age may predict a mild course, their underlying malignancy and receipt of chemotherapy, may increase the risk of severe infection. In adults, hematologic malignancy has been identified as a risk factor for severe COVID outcomes, with early reports describing mortality rates as high as 50% [3, 10, 11]. Leveraging infrastructure developed to ensure comprehensive infection reporting on the currently enrolling, geographically diverse AALL1731 trial, this descriptive analysis provides a disease‐specific estimate of SARS‐CoV‐2 infection outcomes for younger pediatric B‐ALL/LLy in consecutively enrolled patients. In contrast to adults, SARS‐CoV‐2 infection appears to be predominantly mild in young B‐ALL/LLy patients.

In this study, inclusive of 253 patients with SARS‐CoV‐2 infections, only three patients had life‐threatening or fatal infections, with a single death attributable to COVID‐19. More experienced grade 3 infection; however, the CTCAE grading scale, which categorizes “hospitalization or prolongation of existing hospitalization” as grade 3, may overestimate the severity of infection. For example, 20 patients experienced febrile neutropenia with SARS‐CoV‐2 infection, necessitating hospitalization at many institutions regardless of etiology, and thus meeting criteria for grade 3 categorization even if otherwise well.

The large proportion of infections with minimal associated symptoms likely in part reflects the rapid implementation of surveillance and screening testing protocols in pediatric oncology centers. However, the spectrum of disease in this cohort appears even less severe than reported in other pediatric cohorts [5, 12, 13]. Several explanations exist. For one, this population was exclusively composed of patients with NCI SR ALL treated on a frontline trial and does not include children older than 10 who would have been treated on the frontline high‐risk protocol, patients with relapsed leukemia, or those undergoing stem cell transplant who are included in other hematologic malignancy cohorts. This discrepancy highlights the need for disease‐ and therapy‐specific estimates of SARS‐CoV‐2 risk, as substantial heterogeneity exists within oncology populations. In addition, selection and recall bias inherent to registry studies or case reports may skew the severity in other reports higher. Notably, this trend held in DS, a population considered at high risk for severe infection. Patients with DS did not seem to have an increased likelihood of SARS‐CoV‐2 infection or a greater severity of disease among those who contracted it, albeit in a small sample size [14].

This analysis was limited in that it does not capture organ‐specific manifestations of SARS‐CoV‐2 or SARS‐CoV‐2‐related symptom constellations (e.g., multisystem inflammatory syndrome or long‐COVID), nor does it capture COVID‐19 treatment, which may have modified disease severity. Importantly, this cohort largely pre‐dated access to vaccination for children younger than 10, as well as the Omicron variant. As both factors decrease the severity of infection, we anticipate that the tendency toward less severe infection in pediatric B‐ALL/LLy patients will persist as vaccine access expands and virus virulence decreases [15].

Low rates of severe COVID‐19 on AALL1731 support guidance to prioritize treatment of the underlying leukemia, even in the setting of a documented SARS‐CoV‐2 [16, 17]. These data suggest that the burden of SARS‐CoV‐2 may be lower than initially perceived based on data gathered from adults and heterogenous pediatric cohorts. Ongoing surveillance of SARS‐CoV‐2 on COG studies will complement these data through analyses of outcomes in later stages of the pandemic and in other disease‐specific cohorts.

## AUTHOR CONTRIBUTIONS

Caitlin W. Elgarten, Joel C. Thompson, Tamara P. Miller, and Sarah Alexander designed the research study. Research was performed by Caitlin W. Elgarten, Joel C. Thompson, Tamara P. Miller, Sarah Alexander, Mignon L. Loh, Elizabeth A. Raetz, Sumit Gupta, Rachel E. Rau, Anne Angiolillo, and Karen R. Rabin. John A. Kairalla and Cindy Wang performed the data analysis. Susan Conway as one of the people who performed the research. Caitlin W. Elgarten and Sarah Alexander interpreted the data and wrote the manuscript. All authors critically reviewed the manuscript.

## FUNDING INFORMATION

NIH, Grant Numbers: U10 CA98543 (COG Chair's Grant), U10 CA180886 (NCTN Operations Center Grant), U10 CA98413, and U10 CA180899 (COG Statistics and Data Center Grants); St. Baldrick's Foundation; NHLBI Career Development Award, Grant Number: 1K23‐HL161309‐01

## CONFLICT OF INTEREST STATEMENT

The authors declare they have no conflicts of interest.

## ETHICS STATEMENT

These data were collected on AALL1731 (NCT03914625), an ongoing clinical trial approved by the Cancer Therapy and Evaluation Program, the Pediatric Central Institutional Review Board (IRB), and participating center IRBs. Written informed consent and assent (if applicable) are obtained before study entry.

## PATIENT CONSENT STATEMENT

Written informed consent and assent (if applicable) are obtained before study entry.

## Data Availability

Data are available upon reasonable request.
